# Enforced viral replication, a mechanism for immune activation

**DOI:** 10.1186/2047-783X-19-S1-S26

**Published:** 2014-06-19

**Authors:** Karl S Lang, Nadine Honke, Namir Shaabani, Dieter Häussinger, Philipp A Lang

**Affiliations:** 1 Institute of Immunology, Medical Faculty, University of Duisburg-Essen, 45147 Essen, Germany; 2 Clinic of Gastroenterology, Hepatology and Infectious Diseases, Heinrich Heine University, 40225 Düsseldorf, Germany

## 

Since many decades, it is known that viral infection can lead to a strong immune activation. This partially explains the strong association of viral infection with autoimmunity, immunopathology during chronic virus infections and complications during transplantation. One explanation for the strong immune stimulatory effects of viruses is the expression of specific patterns (e.g. viral RNA). Pathogen-specific patterns can be recognized by endogenous receptors (e.g.: Toll -like receptors), which are known to induce a strong innate immune activation. Whether further immunological mechanisms contribute to virus-specific immune activation is current topic of research.

In the recently published work [1,2], we identified the mechanism of enforced viral replication. We found that specialized cells in the spleen and lymph nodes (CD169^+^ macrophages and conventional dendritic cells) selectively allowed replication of viruses. This enforced viral replication led to an exorbitant propagation of viral antigens and viral RNA. Viral antigen led to massive activation of the adaptive immune system, whilst viral RNA activated innate immunity. Therefore the mechanism of enforced viral replication partially explains the association of strong immune activation with virus infection.

First, we analyzed the role of enforced viral replication during infection with cytopathic vesicular stomatitis virus (VSV). We demonstrated that CD169^+^ macrophages express the protein UBP43 (gene name: *Usp18*). UBP43 is a cellular interferon type I (IFN-I) inhibitor. Therefore, due to expression of UBP43, IFN-I could not induce an antiviral status in CD169^+^ macrophages. Thus VSV, once it infected CD169^+^ macrophages, showed strong replication in this particular cell type. Other types of macrophages (Kupffer cells and red pulp macrophages) did not show VSV replication after infection. The enhanced replication of VSV in CD169^+^ macrophages induced a strong immune activation. Genetic depletion of *Usp18* or depletion of CD169^+^ macrophages limited enforced viral replication and resulted in a reduced activation of the adaptive and innate immune response. As a consequence virus could not be controlled resulting in fast death after infection. Together our data show that replication of VSV in specific splenic niches is essential for innate and adaptive immune activation and for surviving an infection with cytopathic VSV.

To investigate the contribution of enforced viral replication to autoimmune disorders we tested its role in the induction of autoimmune diabetes using the RIP-GP mouse model. RIP-GP mice express the glycoprotein of the lymphocytic choriomeningitis virus (LCMV) under the rat insulin promoter. LCMV-specific CD8^+^ T cells, which usually develop during LCMV infection, show cross-reactivity to pancreatic beta cells of RIP-GP mice. Therefore RIP-GP mice develop diabetes during infection with LCMV. We found that induction of LCMV-specific CD8^+^ T cells as well as LCMV induced innate immune response depended on enforced viral replication. Genetic knockdown of *Usp18*, depletion of CD169^+^ macrophages and conventional dendritic cells or pharmacological inhibition of viral life cycle, blunted viral replication in the spleen and limited innate and adaptive immune activation against LCMV. Thereby the development of autoimmune diabetes was prevented.

In summary, professional innate immune cells purposely allow virus replication due to over-expression of UBP43. UBP43 expression and accelerated virus replication was essential to activate the antiviral innate and adaptive immune response. At the one hand this mechanism is absolutely required to protect from fatal virus infection, at the other hand it also strongly contributes to induction of virus induced autoimmune diabetes. In the future, it will be investigated in which other diseases this mechanism could play a role, and whether it may also have clinical implications.
